# Machine learning optimization for hybrid electric vehicle charging in renewable microgrids

**DOI:** 10.1038/s41598-024-63775-5

**Published:** 2024-06-17

**Authors:** Marwa Hassan

**Affiliations:** Arab Academy for Science, Technology and Maritime Transport (AASTMT), Cairo, Egypt 2033, Sheraton,

**Keywords:** Machine learning, Random forest regression, Krill Herd algorithm, Coordinated charging, Intelligent charging, Sustainability, Green energy, Electrical and electronic engineering, Energy storage, Renewable energy

## Abstract

Renewable microgrids enhance security, reliability, and power quality in power systems by integrating solar and wind sources, reducing greenhouse gas emissions. This paper proposes a machine learning approach, leveraging Gaussian Process (GP) and Krill Herd Algorithm (KHA), for energy management in renewable microgrids with a reconfigurable structure based on remote switching of tie and sectionalizing. The method utilizes Gaussian Process (GP) for modeling hybrid electric vehicle (HEV) charging demand. To counteract HEV charging effects, two scenarios are explored: coordinated and intelligent charging. A novel optimization method inspired by the Krill Herd Algorithm (KHA) is introduced for the complex problem, along with a self-adaptive modification to tailor solutions to specific situations. Simulation on an IEEE microgrid demonstrates efficiency in both scenarios. The predictive model yields a remarkably low Mean Absolute Percentage Error (MAPE) of 1.02381 for total HEV charging demand. Results also reveal a reduction in microgrid operation cost in the intelligent charging scenario compared to coordinated charging.

## Introduction

### Problem definition

The operational and managerial aspects of renewable microgrids represent a pivotal frontier in the contemporary pursuit of resilient and sustainable energy infrastructures. These microgrids, leveraging diverse renewable sources like wind, solar, and energy storage, embody the potential for cost-effective, low-emission power generation. However, they are not immune to inherent challenges that demand sophisticated solutions to ensure optimal functionality. A primary challenge stems from the intermittent nature of renewable sources, introducing unpredictability that necessitates advanced control mechanisms. Ensuring a stable and reliable power supply under these conditions becomes a paramount focus, requiring cutting-edge grid management strategies. Furthermore, the integration of energy storage systems and hybrid electric vehicles (HEVs) into the microgrid adds layers of complexity to operational dynamics. Effectively managing HEV charging demands, optimizing energy storage utilization, and orchestrating seamless interactions among various components present intricate challenges. To address these complexities and propel microgrid efficiency, advanced modeling techniques and optimization algorithms are imperative. Striking a delicate balance between energy generation, storage, and consumption is crucial. The overarching goal is not only to enhance operational efficiency but also to minimize environmental impact, contributing to a more sustainable and resilient energy landscape.In light of these challenges, the literature review examines existing methodologies and optimization algorithms in the field of microgrid electric vehicle optimization. By synthesizing prior research, this review aims to identify gaps and opportunities for addressing the complexities associated with renewable microgrids and hybrid electric vehicles.

### Literature review

In recent years, significant advancements have been made in the field of microgrid electric vehicle optimization, addressing numerous challenges and paving the way for more efficient energy management strategies. A multitude of studies have delved into optimal energy management for renewable microgrids, incorporating various elements such as wind units, solar panels, battery storage, and electric vehicles. Thirunavukkarasu et al.^1^ offered a comprehensive review, yet the broad scope may overlook specific limitations of individual methods. Behera and Choudhury^2^ conducted a systematic review, but might not sufficiently address the technical nuances of optimization algorithms. Leonori et al.^3^ introduced Genetic Algorithms, effective but computationally intensive, posing challenges in real-time applications. Dashtaki et al.^4^ tackled uncertainties but may face scalability issues in larger networks. Zhang et al.^5^ proposed a Remora optimization approach, which, while efficient, might struggle with dynamic microgrid conditions. Alamir et al.^6^ developed a pelican optimization technique, which, despite its promise, may lack robustness in balancing objectives. Shezan et al.^7^ evaluated strategies but may not fully account for emerging technologies like electric vehicle integration. Kim and Kim^8^ introduced deep learning, powerful yet resource-intensive. Nallolla et al.^9^ discussed multi-objective algorithms, but scalability and uncertainty considerations remain. Thus, while existing methodologies offer insights, their limitations underscore the necessity for our proposed work to address these gaps and provide a more comprehensive solution for microgrid energy management. Collectively, these studies demonstrate the ongoing efforts to address the challenges and complexities associated with microgrid electric vehicle optimization, contributing to the advancement of sustainable energy systems. Finally, Khorram-Nia et al.^10^ investigated optimal switching in reconfigurable microgrids considering electric vehicles and renewable energy sources, highlighting the importance of adaptive control strategies for dynamic microgrid operations. These studies collectively underscore the ongoing efforts to address the challenges and complexities associated with microgrid electric vehicle optimization, contributing to the advancement of sustainable energy systems. The integration of hybrid wind-solar units has been explored in-depth, revealing promising potential when managed efficiently^11^. To tackle the pervasive issue of uncertainty in renewable microgrids, Eskandari et al.^12^ introduced a stochastic method based on Monte Carlo simulations, bridging the gap between theoretical models and real-world scenarios. Transitioning to data-driven frameworks, Förster et al.^13^ proposed a model utilizing big data for economic and technical decision-making in renewable microgrids. Emphasizing the importance of securing these systems against cyber threats, Aljohani et al.^14^ and Zeng et al.^15^ employed wireless sensor networks and a data intrusion detection approach based on prediction intervals. In the context of energy storage and efficiency enhancement, Chen and Duan^16^ investigated hydrogen production and thermal energy recovery in renewable microgrids, showcasing a potential efficiency increase of 9-18% at peak load. Multi-objective structures optimizing power losses and costs through optimal switching have been explored by Mortaz and Valenzuela^17^ and Tushar et al.^18^, providing valuable insights into improving overall performance. The impact of electric vehicles on microgrid functionality has been assessed, emphasizing the need for accurate modeling of their random behavior^19^. Introducing the vehicle-to-grid (V2G) concept, Taghizadegan et al.^20^ demonstrated its potential to reduce operation costs and create mutually beneficial scenarios. Moreover, Gholami et al.^21^ proposed a risk-oriented energy management strategy for electric vehicle fleets in hybrid AC-DC microgrids. Mohamed et al.^22^ developed a novel fuzzy cloud stochastic framework for energy management of renewable microgrids, maximizing the deployment of electric vehicles. Vitale et al.^23^ utilized dynamic programming for optimal energy management of grid-connected reversible solid oxide cell-based renewable microgrids. Mahesh and Sushnigdha^24^ proposed an improved search space reduction algorithm for optimal sizing of photovoltaic/wind/battery hybrid renewable energy systems, including electric vehicles. Ali et al.^25^ focused on enhancing resilience using mobile electric vehicles in networked microgrids, while Mukhopadhyay et al.^26^ optimized hourly energy scheduling in interconnected renewable microgrids. Salkuti^27^ reviewed advanced technologies for energy storage and electric vehicles, providing insights into their integration. Aybar-Mejía et al.^28^ discussed low-voltage renewable microgrids, focusing on generation forecasting and demand-side management strategies. Mohammadi et al.^29^ proposed a deep learning-based control system for renewable microgrids, aiming to improve system stability and performance. This research landscape underscores the ongoing efforts to develop sophisticated models and optimization techniques for microgrid electric vehicle systems, laying the groundwork for sustainable energy management in the future. Fathima and Palanisamy^30^ explored renewable systems and energy storages for hybrid systems, offering insights into their integration. Norouzi et al.^31^ presented a multi-objective optimal planning framework for electric vehicle charging stations and renewable energy resources in smart microgrids. Thaler et al.^32^ proposed a hybrid model predictive control approach for renewable microgrids and seasonal hydrogen storage. Tan and Chen^33^ addressed multi-objective energy management of multiple microgrids under random electric vehicle charging, aiming to improve overall system efficiency. Vosoogh et al.^34^ developed an intelligent day-ahead energy management framework for networked microgrids, considering high penetration of electric vehicles. Ouramdane et al.^35^ critically reviewed the optimal sizing and energy management of microgrids with vehicle-to-grid technology, identifying future trends. Mohammadi et al.^36^ conducted a comprehensive review of artificial intelligence techniques in microgrids, highlighting their potential applications. Khaleel^37^ discussed intelligent control techniques for microgrid systems, emphasizing their role in enhancing system stability and performance. Mehdi et al.^38^ proposed an artificial intelligence-based nonlinear control strategy for hybrid DC microgrids, focusing on dynamic stability and bidirectional power flow. Lastly, Zulu et al.^39^ provided a comprehensive review of artificial intelligence optimization technique applications in a hybrid microgrid during fault outbreaks, shedding light on their effectiveness in enhancing system resilience. Additionally, recent research papers further highlight the ongoing efforts to advance the field, emphasizing the need for a cohesive and sophisticated model to optimize microgrid electric vehicle systems effectively. Despite the remarkable strides in existing research towards optimizing microgrid electric vehicle systems, a critical gap persists. Many existing studies have offered valuable insights into the optimization of microgrid electric vehicle systems, yet they often lack a unified and comprehensive approach to address the intricate integration of renewable microgrids with the charging demands of hybrid electric vehicles (EVs). Recognizing this gap, this research endeavors to provide a novel perspective that not only rectifies these identified shortcomings but also offers a holistic understanding of sustainable energy management challenges and opportunities for the future. To bridge this gap, our study introduces a groundbreaking integrated framework that harnesses Gaussian Process (GP) regression and the Krill Algorithm to optimize EV charging within renewable microgrids. Recognizing the limitations of existing methodologies in adequately addressing the intricate integration of renewable microgrids with the charging demands of hybrid electric vehicles (EVs), we developed this novel approach to provide a more holistic solution. Unlike previous approaches that may overlook the complex interactions between renewable energy generation, energy storage, and EV charging demands, our methodology confronts these challenges directly. Through GP regression, we accurately model the stochastic behavior of EV charging demands, effectively addressing the inherent uncertainty in renewable microgrid systems. This enables more precise decision-making and enhances system resilience against unpredictable factors. Additionally, GP regression offers the advantage of providing probabilistic predictions, allowing for quantification of uncertainty and risk assessment in decision-making processes. Complementing this, the Krill Algorithm optimizes system performance by considering multiple objectives, including energy efficiency, cost minimization, and grid stability. Its adaptive nature allows for real-time adjustments in response to changing environmental conditions and demand patterns. Moreover, the Krill Algorithm offers the advantage of being inspired by natural behaviors, such as swarm intelligence, which enables efficient exploration of the solution space and robust convergence to optimal solutions. In summary, the proposed approach offers a comprehensive solution to the challenges of hybrid EV integration in renewable microgrids. It not only provides more accurate modeling and better decision-making capabilities but also significantly improves system performance compared to existing methods. By amalgamating the benefits of GP regression and the Krill Algorithm, this research contributes to the advancement of sustainable energy management practices.

### Methodology and tools

This study focuses on integrating the Krill algorithm for microgrid energy management, specifically optimizing Hybrid Electric Vehicle (HEV) charging patterns. Using an IEEE microgrid test system with a hybrid component, historical HEV charging data trains a Gaussian Process Model for predictive analysis. The Krill algorithm plays a crucial role in achieving the dual goals of minimizing operational costs and ensuring a reliable energy supply.The microgrid model comprises of several nodes representing generators, loads, renewable energy sources, and energy storage systems. To ensure dependable power flow and voltage stability, the Newton-Raphson method was chosen. This method was selected due to its well-established efficacy in solving power flow equations and maintaining voltage stability within acceptable thresholds. Its iterative framework enables the computation of steady-state voltage profiles and line flows by iteratively solving a series of nonlinear equations that capture power balance at each node in the microgrid. Through this iterative process and the ability to adjust node voltages until power mismatches meet acceptable criteria, precise computation of voltage magnitudes and phase angles was achieved, thereby satisfying power flow constraints. The six-month simulation assesses accuracy and reliability using metric such as MAPE.This study utilizes Python, a versatile and widely-used programming language in the field of data science, alongside scikit-learn for machine learning and optimization libraries. Python offers a versatile environment, with essential libraries such as NumPy for numerical computations and Pandas for efficient data manipulation. Within the scikit-learn ecosystem, extensive use is made of the GaussianProcessRegressor class to implement Gaussian Process regression, a crucial component for modeling EV charging demands accurately. Moreover, scikit-learn’s optimization modules, including GridSearchCV and RandomizedSearchCV, prove instrumental for fine-tuning hyperparameters and selecting the most suitable models. To ensure optimal model performance, functions from scikit-learn’s preprocessing module, such as StandardScaler and MinMaxScaler, are employed to preprocess input data effectively. The seamless integration of Python and scikit-learn facilitates the development of robust algorithms and the optimization of microgrid energy management with precision and efficiency. Scikit-learn, a powerful machine learning library for Python, offers a comprehensive suite of tools for data preprocessing, model training, and evaluation. Its user-friendly interface and extensive documentation facilitate seamless integration into our research workflow. Furthermore, scikit-learn provides a diverse range of optimization algorithms, enabling us to explore various approaches for fine-tuning our models and achieving optimal performance. The integration of these libraries empowers us to leverage state-of-the-art machine learning techniques and optimization algorithms to address the complex challenges of microgrid electric vehicle optimization. Specifically, scikit-learn enables efficient implementation of Gaussian Process regression for modeling EV charging demands (Eq. (1)). Additionally, Eq. (2) defines the Radial Basis Function (RBF) kernel function used in the model. As for the Krill Herd Algorithm, the equations associated with krill movement and adaptive mechanisms are outlined in Sect. "Main loop", particularly Eqs. (8^8^) through (14).

#### Scalability and adaptability of the proposed model

The scalability of the proposed model to accommodate varying grid sizes is a fundamental aspect that has been diligently considered and addressed in this research. The model is designed to be flexible and adaptable, seamlessly scaling from small-scale microgrids serving localized communities to larger grids spanning expansive geographical areas. This scalability is achieved through several key features. Firstly, the model incorporates modular and hierarchical design principles, enabling it to efficiently handle grids of different sizes and complexities. It breaks down the optimization process into manageable components and layers, allowing it to scale up or down as needed without sacrificing performance or computational efficiency. Additionally, advanced optimization algorithms and techniques, including the utilization of Gaussian Process (GP) and Krill Herd Algorithm (KHA), are employed to manage the increased computational demands associated with larger grid sizes while maintaining high levels of accuracy and robustness. The model also incorporates mechanisms for handling uncertainties in renewable energy generation, fluctuations in energy demand, and dynamic grid conditions, leveraging data-driven approaches and adaptive control strategies to navigate integration challenges commonly encountered in real-world scenarios

#### Uncertainty and variability handling

This study addresses the intricate challenges posed by uncertainty and variability in renewable energy sources within microgrid settings. It presents a meticulously devised methodology that strictly adheres to IEEE standards while harnessing the computational prowess of Python. At the core of this methodology lies the sophisticated utilization of probabilistic modeling techniques. By leveraging historical data on solar radiation and wind speed forecasts, the study analyzes a spectrum of potential scenarios alongside their associated probabilities. This rigorous analysis provides invaluable insights into the variability of renewable energy sources, enabling a deeper understanding and anticipation of energy generation fluctuations. Furthermore, the study advocates for the adoption of scenario-based analysis, departing from conventional deterministic methods. Through the evaluation of an extensive array of potential outcomes, each characterized by varying levels of solar irradiance or wind speed, the methodology empowers microgrid operators to devise robust strategies for optimizing performance and mitigating risks associated with renewable energy fluctuations. Parameter selection in scenario-based analysis is refined through sensitivity analysis techniques, enabling the customization of strategies to suit diverse operating conditions and ensuring adaptability and resilience in uncertain environments. Additionally, the study seamlessly integrates advanced stochastic optimization techniques, leveraging stochastic dynamic programming within the Newton-Raphson method. This integration explicitly addresses uncertainty within optimization processes, facilitating the development of robust operational strategies that consistently excel across diverse scenarios. Optimal parameter values for stochastic dynamic programming are meticulously determined through extensive experimentation, with sensitivity analysis and performance evaluation metrics employed to assess the impact of parameter variations on optimization effectiveness. Moreover, the study advocates for the strategic integration of hybrid energy systems, representing a novel approach to mitigating individual energy source variability. By judiciously combining multiple renewable energy sources with complementary characteristics, such as solar and wind power, the approach maximizes system resilience and reliability. Techno-economic analyses in hybrid energy system integration consider factors such as resource availability, cost, and environmental impact, ensuring optimal utilization of each energy source and enhancing microgrid resilience amidst renewable energy output variability. In summary, this meticulously crafted framework represents a significant advancement in microgrid management, providing a comprehensive solution to uncertainty and variability in renewable energy sources.

### Paper structure

This manuscript is meticulously organized to provide a comprehensive examination of microgrid energy management challenges and opportunities. The structure begins with an introductory section, setting the stage for understanding the context and significance of the study. Following this, the second section dives into the development of an innovative Machine Learning-Based Energy Management Framework, shedding light on the AI model utilized and its role in addressing microgrid complexities. The subsequent section delves into HEV modeling intricacies, offering a detailed exploration of this pivotal component within microgrid systems. Transitioning smoothly, the fourth section meticulously lays out the problem setup, elucidating the objective function and constraints that guide the study’s methodologies. Building upon this foundation, the fifth section presents the simulation and results, offering readers a thorough analysis of the outcomes derived from the applied techniques. Lastly, the paper concludes by synthesizing key findings and implications drawn from the multifaceted exploration conducted, thereby offering insights that contribute to advancing the field of microgrid energy management.

## Development of a machine learning-based energy management framework

This section comprises two primary components: a modeling approach utilizing Gaussian Process models, and an optimization strategy employing the Krill Herd Algorithm (KHA). The subsequent sections delve into the details of each of these components and their applications within the framework.

### Gaussian process model for electric vehicle charging demand

The Gaussian Process Model is an advanced approach for uncovering the underlying relationships between input and output variables within a system. Unlike traditional AI-based models that aim to discover unknown relationships between inputs and outputs, Gaussian Process Models take into account model complexity while minimizing training errors, providing a more nuanced approach to modeling. In the context of electric vehicle charging demand and considering constraints and a trade-off parameter *C*, this Gaussian Process model can be represented as follows

1 $$\begin{aligned} f(x) \sim \text {GP}(\mu _{\text {nonlinear}}(x), k_{\text {RBF}}(x, x')) \end{aligned}$$

where:

$$\begin{aligned} f(x)&: \text {Predicted charging demand for input } x \\ \mu _{\text {nonlinear}}(x)&: \text {Nonlinear mean function representing the expected charging demand} \\ k_{\text {RBF}}(x, x')&: \text {Radial Basis Function (RBF) kernel function} \end{aligned}$$

The mean function represents the expected charging demand and can be a nonlinear function of input variables. It characterizes the trend of the data.

#### RBF Kernel function $k_{\text {RBF}}(x, x')$

The Radial Basis Function (RBF) kernel captures smooth and continuous variations in charging demand data. It is defined as:

2 $$\begin{aligned} k_{\text {RBF}}(x, x') = \exp \left( -2\sigma ^2 \Vert x - x' \Vert ^2\right) \end{aligned}$$

where $\sigma$ is the standard deviation of the kernel, controlling the smoothness of the function.

#### Constraints

The constraints ensure that the model respects the training data and maintains a trade-off between complexity and training error. The constraints are as follows:

3 $$\begin{aligned}{} & f(x_i) - \mu _{\text {nonlinear}}(x_i) - \beta - y_i + \mu _{\text {nonlinear}}(x_i) + \beta \le \xi _i^* \\&\xi _i^* \ge 0 \quad \text {for } i = 1,2,\ldots ,n \end{aligned}$$

4 $$\begin{aligned}{} &f(x_i) - \mu _{\text {nonlinear}}(x_i) - \beta - y_i + \mu _{\text {nonlinear}}(x_i) + \beta \le \epsilon + \xi _i \\&\xi _i \ge 0 \quad \text {for } i = 1,2,\ldots ,n \end{aligned}$$

where:

$$\begin{aligned} & y_i : \text {Observed charging demand for the} i-\text {th data point} \\ & \beta : \text {Parameter} \\ & \epsilon : \text {Loss function parameter penalizing high training errors} \\ & \xi_i^*, \xi _i : \text {Slack variables} \end{aligned}$$

#### Optimization objective

To solve for the model parameters, including the kernel hyperparameters, mean function parameters, and trade-off parameter *C*, we need to minimize the following objective:

5 $$\begin{aligned} \min _{\mu _{\text {nonlinear}}, \beta , \xi _i^*, \xi _i} \left[ \frac{1}{2} \left( \mu _{\text {nonlinear}}^T \mu _{\text {nonlinear}} + C \sum _{i=1}^{n} (\xi _i^* + \xi _i)\right) \right] \end{aligned}$$

Subject to the defined constraints.

#### Predictions

The predictions for charging demand at a new point *x* are based on the model’s mean function:

6 $$\begin{aligned} {\hat{f}}(x) = \mu _{\text {nonlinear}}(x) \end{aligned}$$

where ${\hat{f}}(x)$ is the predicted charging demand at *x*.

#### Uncertainty estimation

The uncertainty in the predictions can be estimated using the RBF kernel:

7 $$\begin{aligned} \text {Var}[{\hat{f}}(x)] = k_{\text {RBF}}(x, x') \end{aligned}$$

In selecting appropriate values for the parameters $x$ and $x'$ within the Radial Basis Function kernel, meticulous consideration is paramount. These values should be chosen to comprehensively represent charging scenarios while balancing similarity and diversity to bolster model performance and generalization. Furthermore, understanding the impact of different values on the overall performance is critical for optimizing model accuracy and robustness. For instance, selecting smaller values for $x$ and $x'$ in the Radial Basis Function (RBF) kernel might lead to smoother predictions with reduced variance but could potentially overlook intricate patterns in the charging demand data. Conversely, larger values for these parameters might capture more complex variations in the data but could result in overfitting and decreased generalization to unseen data points. By systematically exploring various combinations of $x$ and $x'$ values across a range of scenarios and datasets, researchers can gain insights into the trade-offs between model complexity, predictive accuracy, and computational efficiency. This iterative process of experimentation and analysis is essential for fine-tuning the Gaussian Process Model and optimizing its performance for diverse electric vehicle charging demand scenarios.

### Optimization technique

Testing various techniques, particularly those inspired by natural algorithms like the Krill Herd Algorithm (KHA), is crucial for fine-tuning the setting parameters of C and $\sigma$ in the Gaussian Process Model, ensuring accurate predictions in electric vehicle charging demand modeling. This comprehensive evaluation helps identify the most effective optimization strategy, enhancing the model’s predictive performance.

#### Krill Herd algorithm (KHA)

Initialize Krill Individuals: Generate an initial population of krill individuals, each represented as ${{\mathcal {K}}}_i$ with a position in the search space and associated GRP parameters *C* and $\sigma$. Initialize their positions randomly within the search space:

8 $$\begin{aligned} \theta _i, \phi _i \in [\theta _{\text {min}}, \theta _{\text {max}}] \times [C_{\text {min}}, C_{\text {max}}] \times [\sigma _{\text {min}}, \sigma _{\text {max}}] \end{aligned}$$

where $\theta _{\text {min}}, \theta _{\text {max}}, C_{\text {min}}, C_{\text {max}}, \sigma _{\text {min}}, \sigma _{\text {max}}$ are the minimum and maximum values for $\theta$, *C*, and $\sigma$.

Evaluate Objective Function:

Evaluate the fitness of each krill by applying GRP with the associated $C_i$ and $\sigma _i$ values to the dataset. Calculate the Mean Absolute Percentage Error (MAPE) as the fitness value, representing prediction accuracy:

9 $$\begin{aligned} \text {MAPE}({{\mathcal {K}}}_i) = \frac{1}{N} \sum _{j=1}^{N} \left| \frac{y_j - {\hat{y}}_j}{y_j} \right| \times 100\% \end{aligned}$$

where *N* is the number of data points, $y_j$ is the actual target value, and ${\hat{y}}_j$ is the predicted value using GP with ${{\mathcal {K}}}_i$.

#### Main loop

Krill Movement:

Separation Movement:

Krill individuals tend to maintain a minimum separation distance from each other. Calculate the new position $\theta _i(t+1)$ based on the separation factor:

10 $$\begin{aligned} \theta _i(t+1) = \theta _i(t) + \Delta \theta _i(t+1) \end{aligned}$$

where

$$\begin{aligned} \Delta \theta _i(t+1) = \Delta s \sum _{j=1}^{N} d(\theta _i(t), \theta _j(t)) (\theta _i(t) - \theta _j(t)) \end{aligned}$$

Alignment Movement:

Krill adjust their speeds to align with neighboring krill. Calculate the new position $\theta _i(t+1)$ based on the alignment factor:

11 $$\begin{aligned} \theta _i(t+1) = \theta _i(t) + \Delta \theta _i(t+1) \end{aligned}$$

where

$$\begin{aligned} \Delta \theta _i(t+1) = \Delta a \sum _{j=1}^{N} (\phi _j(t) - \phi _i(t)) (\theta _j(t) - \theta _i(t)) \end{aligned}$$

Cohesion Movement:

Krill move toward the center of mass of the population. Calculate the new position $\theta _i(t+1)$ based on the cohesion factor:

12 $$\begin{aligned} \theta _i(t+1) = \theta _i(t+1) + \Delta \theta _i(t+1) \end{aligned}$$

where

$$\begin{aligned} \Delta \theta _i(t+1) = \Delta c \frac{1}{N} \sum _{j=1}^{N} (\theta _j(t) - \theta _i(t)) \end{aligned}$$

Attraction Movement:

Krill move toward areas of higher food concentration (improved GRP performance). Calculate the new position $\theta _i(t+1)$ based on the attraction factor:

13 $$\begin{aligned} \theta _i(t+1) = \theta _i(t+1) + \Delta \theta _i(t+1) \end{aligned}$$

where

$$\begin{aligned} \Delta \theta _i(t+1) = \Delta f (F_{\text {loc}} - \theta _i(t)) \end{aligned}$$

Distraction Movement:

Krill react to threats (less promising solutions) by moving away. Calculate the new position $\theta _i(t+1)$ based on the distraction factor:

14 $$\begin{aligned} \theta _i(t+1) = \theta _i(t+1) + \Delta \theta _i(t+1) \end{aligned}$$

where

$$\begin{aligned} \Delta \theta _i(t+1) = \Delta e (E_{\text {loc}} + \theta _i(t)) \end{aligned}$$

In optimizing the Gaussian Process Model for electric vehicle charging demand modeling, selecting parameter values in the Krill Herd Algorithm (KHA) is pivotal for accurate predictions and efficient optimization. Parameter choices, including the separation ($\Delta s$), alignment ($\Delta a$), cohesion ($\Delta c$), attraction ($\Delta f$), and distraction ($\Delta e$) factors, play a crucial role in achieving this balance between exploration and exploitation. Prioritizing values that promote both exploration and exploitation ensures adequate exploration of the solution space while exploiting promising regions. This prevents premature convergence to suboptimal solutions while facilitating convergence towards the global optimum. Furthermore, parameter values are tailored to the characteristics of the data, such as its complexity, size, and variability. For instance, smaller values may be preferred in scenarios with high variability or sparse data to encourage more exploration, while larger values may be suitable in scenarios with well-defined patterns or abundant data to emphasize exploitation and refine solutions efficiently

#### Adaptive mechanisms

In the final stages of the optimization process, inspired by the Krill Algorithm, several crucial steps are taken to fine-tune parameters and achieve an optimal solution. Initially, parameters $\Delta s$, $\Delta a$, $\Delta c$, $\Delta f$, and $\Delta e$ are dynamically adjusted using adaptive formulas, drawing inspiration from the Krill Algorithm’s adaptability.

Following parameter adjustment, the optimization process involves updating the best solution identified thus far, guided by the Mean Absolute Percentage Error (MAPE) performance metric. This ensures that promising solutions are retained and leveraged to enhance overall optimization.

Conversely, the worst solution encountered during the optimization process is utilized as a reference point, serving as a measure of the adversary in the pursuit of an optimal outcome. This dual evaluation approach enables the optimization process to strike a balance between exploring potential improvements and avoiding less favorable solutions.

**Figure 1:**
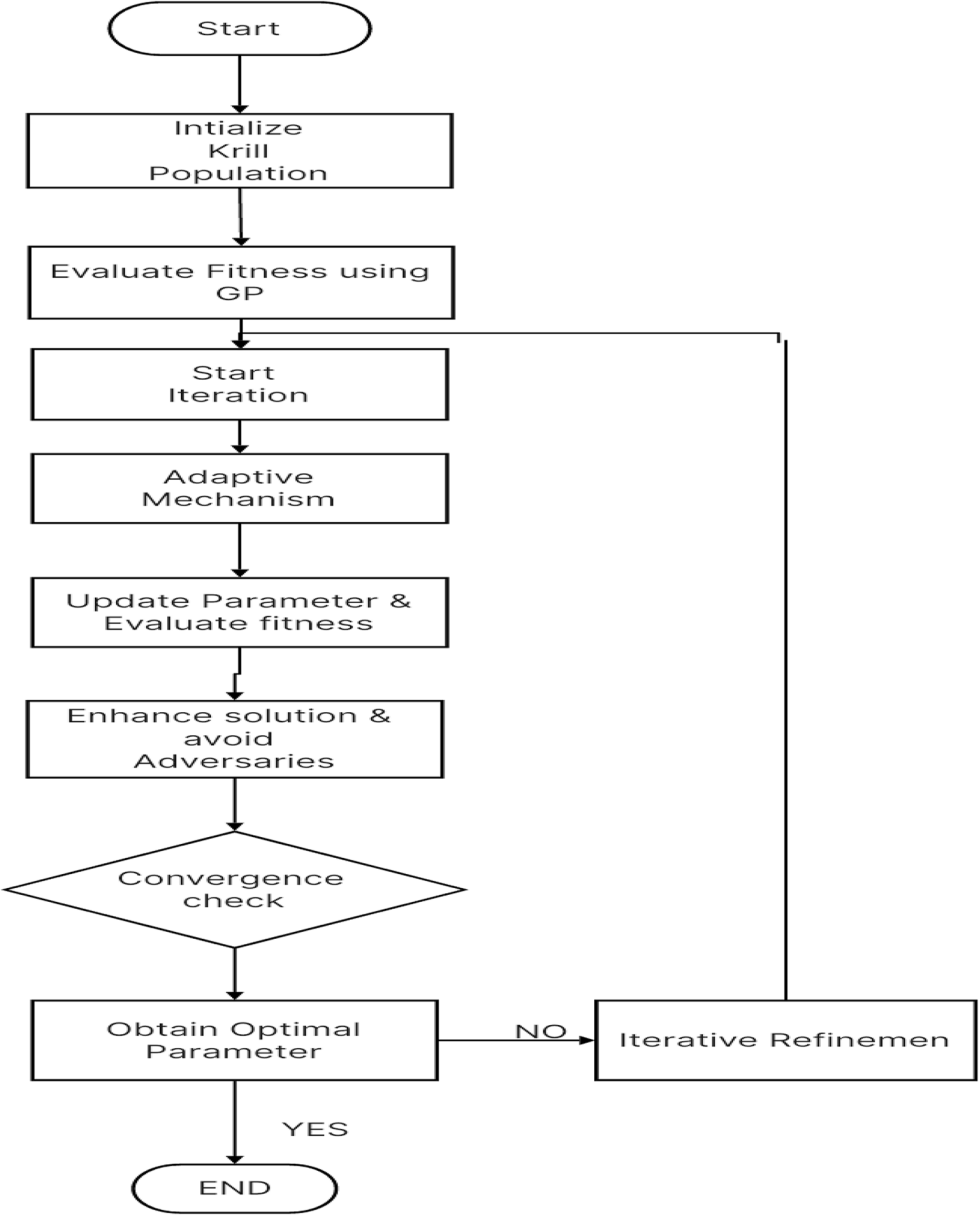
Flow chart of the proposed system.

These iterative optimization steps, encompassing parameter adjustment, solution enhancement, and adversary avoidance, are reiterated in a cyclical manner. This iterative process continues for a defined number of iterations, denoted as MaxIter, or until convergence is attained based on a predefined stopping criterion. This dynamic and adaptive approach, inspired by the Krill Algorithm, contributes to the progressive refinement of the solution space, ultimately converging towards an optimal outcome. Figure 1 depicts the flowchart outlining the proposed system.

## HEV charging demand modeling

The charging behavior of Hybrid Electric Vehicles (HEVs) is influenced by various factors, including market share, state of charge (SoC), charging duration, and more. To comprehensively account for the impact of HEVs on the system, it is essential to define these uncertain variables with precision, reducing system variability. This paper Gaussian Process Mode for predicting the overall charging demands of HEVs.

### Charging modeling of HEVs

The charging behavior of Hybrid Electric Vehicles (HEVs) is influenced by various factors, including market share, State of Charge (SoC), charging time and duration, and more. To incorporate HEVs’ effects into the system, it’s essential to define these uncertain factors accurately, reducing randomness in the system. This paper utilizes the Gaussian Process Mode method to predict the overall charging demands of HEVs.

**Figure 2:**
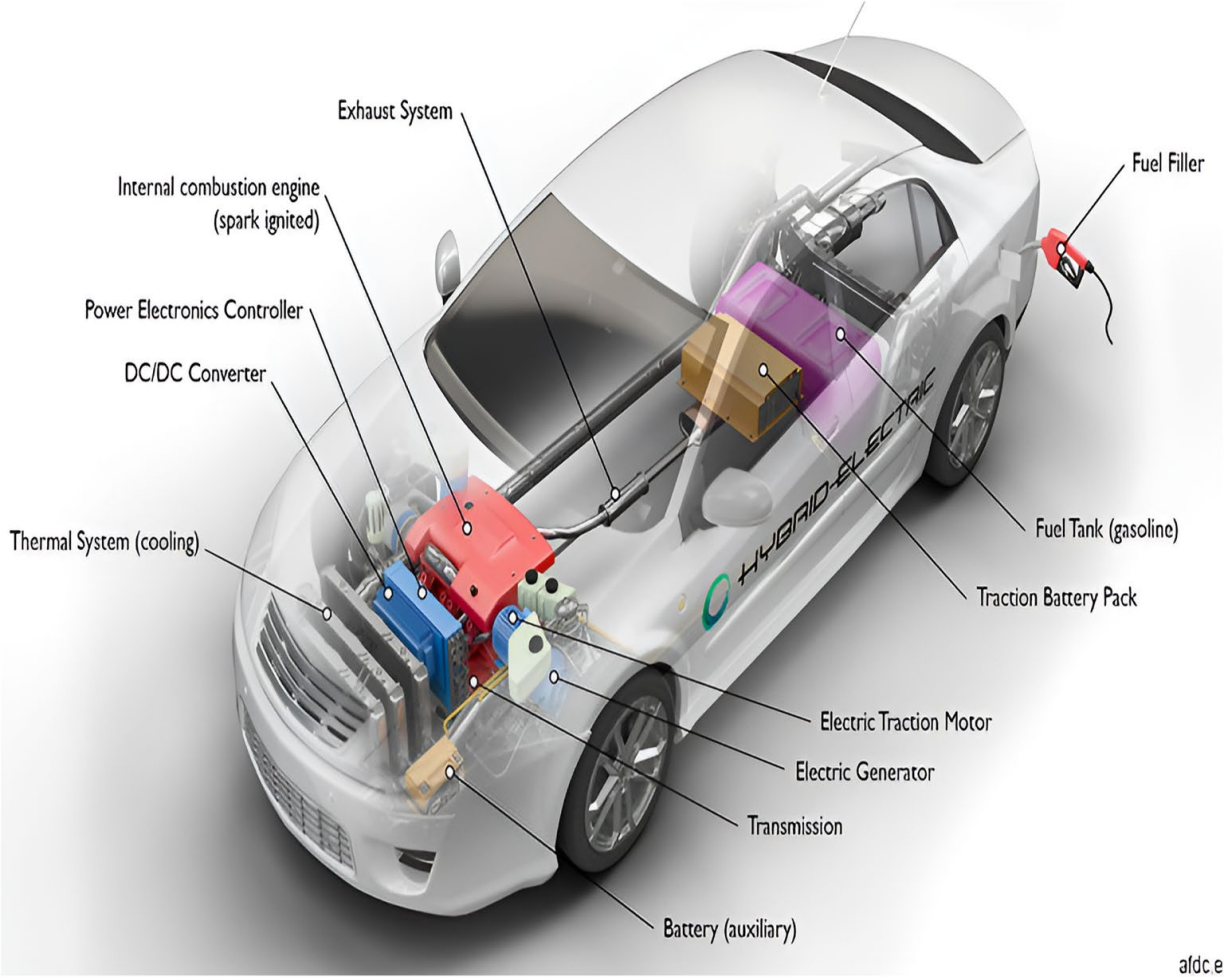
Key elements in a standard hybrid electric vehicle (HEV)^40^.

From a technical perspective, HEVs can source energy from either gasoline or electricity. Figure 2 illustrates the main components in HEVs^40^.

There is a need to understand the distribution of charging in these vehicles. Two strategies are considered: Coordinated Charging and Intelligent Charging.

In Coordinated Charging, HEVs are allowed to initiate charging during specific hours based on customers’ daily patterns. This typically occurs during off-peak hours, around 6 p.m. to 7 p.m. The charging initiation function is defined as follows:

15 $$\begin{aligned} f(t_{\text {start}}) = {\left\{ \begin{array}{ll} 1 &{} \text {if } \gamma _1 \le t_{\text {start}} \le \gamma _2 \\ 0 &{} \text {otherwise} \end{array}\right. } \end{aligned}$$

where $$\gamma _1 = 18$$ and $$\gamma _2 = 19$$.

The second strategy is Intelligent Charging, where vehicles charge based on the microgrid’s electrical load curve and power companies’ bidding offers. This strategy is modeled using a normal distribution function:

16 $$\begin{aligned} f(t_{\text {start}}) = \frac{1}{\sigma \sqrt{2\pi }} e^{-\frac{1}{2}\left( \frac{t_{\text {start}} - \mu }{\sigma }\right) ^2} \end{aligned}$$

where $$\mu = 1$$ and $$\sigma = 3$$.

Once the starting time is known, the charging demand of HEVs can be determined. The average mileage of each vehicle can be estimated as follows:

17 $$\begin{aligned} f(m) = {\left\{ \begin{array}{ll} \sqrt{\frac{e^{-(\ln (m)-\mu )^2}}{2\sigma ^2}} &{} \text {if } m > 0 \\ 0 &{} \text {if } m \le 0 \end{array}\right. } \end{aligned}$$

With the estimated mileage (*m*), the State of Charge (SoC) of the battery is determined using a straightforward equation:

18 $$\begin{aligned} \text {SoC} = {\left\{ \begin{array}{ll} 100\% &{} \text {if } m \le \text {ER} \\ 100\% - \frac{m}{\text {ER}} &{} \text {if } m > \text {ER} \end{array}\right. } \end{aligned}$$

where ER represents the total electric range, and exceeding this range results in the vehicle shutting down.

Considering SoC and battery capacity ($C_{\text {bat}}$), the charging duration can be estimated as follows:

19 $$\begin{aligned} t_{\text {D}} = \frac{C_{\text {bat}} \cdot (1 - \text {SoC}) \cdot \text {DOD}}{\eta _{\text {c}} \cdot P_{\text {c}}} \end{aligned}$$

where $P_{\text {c}}$ is the charger power and $\eta _{\text {c}}$ represents the charging efficiency. Tables 1 and 2 provide information about four charger types corresponding to four different classes of HEVs. This data will be used in subsequent simulation results for a comprehensive analysis.

**Table 1:** Charger types in HEVs.

Charger type	Input voltage	Maximum power (kW)
Level 1	120 VAC	1.44
Level 2	208-240 VAC	11.5
Level 3	208-240 VAC	96
Level 3 (DC)	208-600 VDC	240

**Table 2:** HEV classes.

Class	Market share	Min $C_{\text {bat}}$ (kWh)	Max $C_{\text {bat}}$ (kWh)
Micro car	0.2	8	12
Economy car	0.3	10	14
Mid-Size car	0.3	14	18
SUV	0.3	19	23

The market share of HEVs is randomly determined based on their type/class (Table 2) with a normal distribution function with characteristics of mean and standard deviation as below:

20 $$\begin{aligned} \mu _{C_{\text {bat}}}= & {} \frac{\text {Min}C_{\text {bat}} + \text {Max}C_{\text {bat}}}{2} \end{aligned}$$

21 $$\begin{aligned} \sigma _{C_{\text {bat}}}= & {} \frac{\text {Max}C_{\text {bat}} - \text {Min}C_{\text {bat}}}{2} \end{aligned}$$

## Problem setup

In the realm of energy management, the primary responsibility of the operator revolves around ensuring the dependable and secure supply of energy to electrical consumers, all while striving to minimize expenses . As a result, the objective function combines operational costs and technical expenditures, manifesting in the following manner Minimize the total cost, considering both operating and technical costs:

22 $$\begin{aligned} \begin{aligned}{}&\text {Minimize the total cost, considering both operating and technical costs:} \\&\text {MinimizeCost} = \\&\sum _{\forall \omega _b} \sum _{\forall \tau _t} \left( \rho _G \cdot \text {OperatingCost}_{G,\omega _b,\tau _t} + \rho _M \cdot \text {OperatingCost}_{M,\omega _b,\tau _t} \right. \\&\left. + \rho _{MPHEV} \cdot \text {EVChargingCost}_{\omega _b,\tau _t} + \rho _{MR} \cdot \text {TechnicalCost}_{\omega _b,\tau _t} \right) \\&+ \lambda \cdot \text {PenaltyCost} \end{aligned} \end{aligned}$$

### Operating cost constraints for distributed generators (DGs)

#### Active power limits

23 $$\begin{aligned} P_{\text {min},m,t} \le P_{G,m,t} \le P_{\text {max},m,t} \quad \forall m \in \Omega _B, \forall t \in \Omega _T \end{aligned}$$

#### Reactive power limits

24 $$\begin{aligned} Q_{\text {min},m,t} \le Q_{G,m,t} \le Q_{\text {max},m,t} \quad \forall m \in \Omega _B, \forall t \in \Omega _T \end{aligned}$$

#### Ramp Up/Down limits

25 $$\begin{aligned}{} & {} P_{G,m,t} - P_{G,m,t-1} \le R_{U,m} P_{G,m,t-1} \quad \forall m \in \Omega _B, \forall t \in \Omega _T \end{aligned}$$

26 $$\begin{aligned}{} & {} P_{G,m,t-1} - P_{G,m,t} \le R_{D,m} P_{G,m,t} \quad \forall m \in \Omega _B, \forall t \in \Omega _T \end{aligned}$$

### Constraints related to energy storage systems

#### Minimum charging time

27 $$\begin{aligned} T_{\text {Ch}} \ge CTS(y_{\text {Ch},m,t} - y_{\text {Ch},m,t-1}) \quad \forall m \in \Omega _{\text {BS}}, \forall t \in \Omega _T \end{aligned}$$

#### Minimum discharging time

28 $$\begin{aligned} T_{\text {Disch}} \ge DTS(y_{\text {Disch},m,t} - y_{\text {Disch},m,t-1}) \quad \forall m \in \Omega _{\text {BS}}, \forall t \in \Omega _T \end{aligned}$$

#### Charging and discharging mode constraints

29 $$\begin{aligned} y_{\text {Ch},m,t} + y_{\text {Disch},m,t} \le 1 \quad \forall m \in \Omega _{\text {BS}}, \forall t \in \Omega _T \end{aligned}$$

### Constraints related to adjustable load demand

30 $$\begin{aligned} PAD_{zAD,m,t} \le PD_{m,t} \le PAD_{zAD,m,t} \quad \forall m \in \Omega _{BAD}, \forall t \in \Omega _T \end{aligned}$$

### Constraints related to DGs and energy storage systems

#### Minimum up and down time limits for DGs

31 $$\begin{aligned}{} & {} T_{G,\text {on}} \ge UTG(x_{G,m,t}) \quad \forall m \in \Omega _{BG}, \forall t \in \Omega _T \end{aligned}$$

32 $$\begin{aligned}{} & {} T_{G,\text {off}} \ge DTG(x_{G,m,t}) \quad \forall m \in \Omega _{BG}, \forall t \in \Omega _T \end{aligned}$$

### Constraints for total energy stored in batteries

#### Total energy stored in batteries

33 $$\begin{aligned} CS_{m,t}= & {} CS_{m,t-1} + \eta _{\text {Disch}} P_{\text {Disch},m,t} - \eta _{\text {Ch}} P_{\text {Ch},m,t} \quad \forall m \in \Omega _{\text {BS}}, \forall t \in \Omega _T \end{aligned}$$

34 $$\begin{aligned}{} & {} C_{mS,t} \le CS_{m,t} \le C_{mS,t} \quad \forall m \in \Omega _{\text {BS}}, \forall t \in \Omega _T \end{aligned}$$

### Constraints for bus voltage and main grid power limits

#### Bus voltage limits

35 $$\begin{aligned} V_{m,t} \le V \le V_{m,t} \quad \forall m \in \Omega _B, \forall t \in \Omega _T \end{aligned}$$

#### Main grid power limits

36 $$\begin{aligned} -P_M \le P_{M,m,t} \le P_M \quad \forall m \in \Omega _B, \forall t \in \Omega _T \end{aligned}$$

### Constraints for reconfiguration using remote switches

#### Binary variables indicating the status of lines

37 $$\begin{aligned} 0 \le I_{mLn,t} \le I_{LmLn,t} \quad \forall m,n \in \Omega _L, \forall t \in \Omega _T \end{aligned}$$

38 $$\begin{aligned} \sum _{\begin{array}{c} mn \in \Omega _L \\ m\in \Omega _B \end{array}} I_{mLn,t} = 1 \quad \forall m \in \Omega , \forall t \in \Omega _T \end{aligned}$$

39 $$\begin{aligned}{} & {} 0 \le \theta _{mn,t} \le w_{mn,t} \quad \forall mn \in \Omega , \forall t \in \Omega _T \end{aligned}$$

40 $$\begin{aligned}{} & {} P_{\text {min},m,t} \le P_{G,m,t} \le P_{\text {max},m,t} \quad \forall m \in \Omega _B, \forall t \in \Omega _T \end{aligned}$$

Electric vehicle

41 $$\begin{aligned} 0&\le P_{EV,\omega _b,\tau _t} \le P_{\text {max, EV},\omega _b} \quad \forall \omega _b, \forall \tau _t \end{aligned}$$

Voltage limit

42 $$\begin{aligned} V_{\text {min},m,t}&\le V_{m,t} \le V_{\text {max},m,t} \quad \forall m \in \Omega _B, \forall t \in \Omega _T \end{aligned}$$

Current limit

43 $$\begin{aligned} I_{\text {min},\omega _l,\tau _t}&\le I_{\omega _l,\tau _t} \le I_{\text {max},\omega _l,\tau _t} \quad \forall \omega _l, \forall \tau _t \end{aligned}$$

44 $$\begin{aligned} \text {PenaltyCost}&\ge \sum _{\text {violations}} \text {PenaltyWeight} \cdot \text {Deviation} \end{aligned}$$

The charging patterns of Hybrid Electric Vehicles (HEVs) are influenced by a multitude of factors, including market penetration, State of Charge (SoC), charging duration, and more. To seamlessly integrate HEVs into the system, it is crucial to precisely define these uncertain parameters, thereby reducing inherent variability. This study employs the Support Vector Regression (SVR) method to predict the overall charging demands of HEVs.

From a technical perspective, HEVs are versatile in their ability to draw energy from either traditional gasoline or electricity sources. Figure 2 offers an overview of the fundamental components of HEVs^35^. In this representation, the cylindrical component signifies fuel storage, emphasizing that HEVs operate by harnessing power from both electrical and fossil fuel sources. To extend this discussion to microgrid energy management, it’s essential to consider how constraint parameters are selected to optimize system performance and stability.In this study, the selection of constraint parameters was methodically driven by a deep understanding of the microgrid’s unique characteristics and operational needs. The research team began by closely examining the size and complexity of the microgrid, alongside the types of distributed energy resources (DERs) integrated within it. This analysis allowed for the tailoring of constraints to match the capabilities of the specific DERs, ensuring they could effectively meet demand without jeopardizing system stability. For instance, when defining the Active and Reactive Power Limits for distributed generators (DGs), meticulous consideration was given to each generator’s capacity and operational behavior. By aligning limits with the capabilities of these generators, the aim was to strike a balance between meeting demand and preventing system overload. The approach to Energy Storage System parameters involved a thorough assessment of storage capacity and anticipated load fluctuations. By carefully selecting charging and discharging times, the goal was to optimize energy storage utilization while minimizing disruptions to grid operations. Adjustable Load Demand constraints were chosen to provide the desired level of flexibility in managing load demands, all while ensuring compatibility with available generation capacity. This involved a nuanced understanding of the microgrid’s load profile and resource capabilities. Similarly, Bus Voltage and Main Grid Power Limits were tailored to maintain voltage stability and manage power flow within acceptable thresholds, accounting for the microgrid’s specific infrastructure and operational needs. Finally, parameters governing Reconfiguration Using Remote Switches were designed to enhance the microgrid’s reliability and resilience, enabling efficient adaptation to changing conditions

### Power flow analysis and voltage stability

The power flow equations within a microgrid are vital for ensuring reliable operation and voltage stability. They are typically addressed using iterative methods such as the Newton-Raphson approach. One key equation is the power balance equation at each node, which maintains overall power balance by equating injected and withdrawn power. Mathematically, this equation ensures that the sum of injected active and reactive power equals the sum of withdrawn power at each node:

45 $$\begin{aligned} P_{i\text {injected}} - P_{i\text {withdrawn}} + j(Q_{i\text {injected}} - Q_{i\text {withdrawn}}) = 0 \end{aligned}$$

where $P_{i\text {injected}}$ and $Q_{i\text {injected}}$ represent the injected active and reactive power at node *i*, and $P_{i\text {withdrawn}}$ and $Q_{i\text {withdrawn}}$ represent the withdrawn active and reactive power at node *i*. Another critical aspect of power flow analysis is the relationship between voltage magnitudes and phase angles at neighboring nodes. This relationship governs power flow across transmission lines and ensures voltage stability within the system. Mathematically, the relationship between voltage magnitudes and phase angles at node *i* and its neighboring node *j* can be expressed as:

46 $$\begin{aligned} V_i\angle \theta _i - V_j\angle \theta _j = Z_{ij}(I_{ij}) \end{aligned}$$

where $V_i$ and $V_j$ are the voltage magnitudes at nodes *i* and *j*, $\theta _i$ and $\theta _j$ are the phase angles at nodes *i* and *j*, $Z_{ij}$ is the impedance of the transmission line connecting nodes *i* and *j*, and $I_{ij}$ is the complex current flowing from node *i* to node *j*. The Newton-Raphson method is employed to iteratively solve these nonlinear equations, adjusting node voltages until power mismatches meet acceptable criteria. By doing so, it ensures balanced power distribution and maintains voltage stability within acceptable thresholds, addressing operating security concerns. The study’s methodology addresses challenges related to power flow constraints, including fluctuating demand and intermittent renewable energy sources, by integrating advanced optimization techniques into the Newton-Raphson algorithm. By doing so, the study aims to enhance the resilience and efficiency of microgrid operation in the face of dynamic energy demands and environmental variability. In the context of microgrid energy management, the selection of parameters for power flow analysis and voltage stability is crucial for ensuring reliable operation and system resilience. When determining these values, various factors specific to the microgrid’s characteristics and operational requirements must be considered. Firstly, the impedance values ($Z_{ij}$) of transmission lines connecting different nodes within the microgrid are chosen based on factors such as line length, material, and loading conditions. These values directly influence the relationship between voltage magnitudes and phase angles, as described by Equation (46). Selecting appropriate impedance values ensures efficient power transfer and voltage stability across the microgrid. Additionally, the criteria for acceptable power mismatches and voltage thresholds are established to maintain system stability under varying operating conditions. These criteria are determined based on factors such as load variations, renewable energy generation fluctuations, and grid disturbances. Optimizing these criteria involves striking a balance between system stability and operational efficiency. Furthermore, the selection of parameters for the Newton-Raphson method, such as convergence criteria and iteration limits, plays a crucial role in the accuracy and efficiency of power flow analysis. Setting appropriate values for these parameters ensures that the iterative solution converges to a stable solution within a reasonable computational time. The impact of these parameter selections on overall performance is significant. For example, overly conservative impedance values may result in excessive voltage drops and power losses, leading to reduced system efficiency. Conversely, overly aggressive convergence criteria may increase computational burden without significant improvement in accuracy.

### Limitations

While the proposed methodology represents a significant advancement in microgrid management, it is essential to acknowledge certain limitations inherent in its components. One potential limitation arises from the computational complexity associated with solving power flow equations using the Newton-Raphson method. Although effective for voltage stability analysis and power flow optimization in steady-state conditions, the iterative nature of this approach demands significant computational resources, particularly in large microgrid networks or real-time applications. Despite advancements in computing technology, managing these computational demands remains a consideration for practical implementation. Similarly, the Krill algorithm’s sensitivity to parameter settings and its reliance on underlying assumptions pose another limitation. Suboptimal parameter choices or deviations from assumed model dynamics may lead to subpar performance or convergence issues. However, conducting sensitivity analysis and careful parameter calibration during algorithm development can mitigate this limitation to a large extent. Additionally, the Gaussian Process Model’s effectiveness relies on the assumption of stationarity in underlying data distributions. In real-world scenarios where renewable energy dynamics exhibit non-stationary behavior, predictive accuracy may be compromised. Techniques such as kernel selection and hyperparameter tuning can enhance the model’s adaptability, but addressing non-stationarity remains a consideration. Moreover, integrating advanced optimization techniques like stochastic dynamic programming introduces additional computational overhead. While these techniques enhance robustness and resilience to uncertainty, they also extend optimization timeframes. However, with careful algorithm design and optimization, these computational demands can be managed effectively without compromising performance. In summary, while the methodology presents several limitations, such as computational complexity and sensitivity to parameter settings, addressing these challenges through careful analysis and algorithmic optimization ensures its continued effectiveness in microgrid management.

## Simulation and results

**Figure 3:**
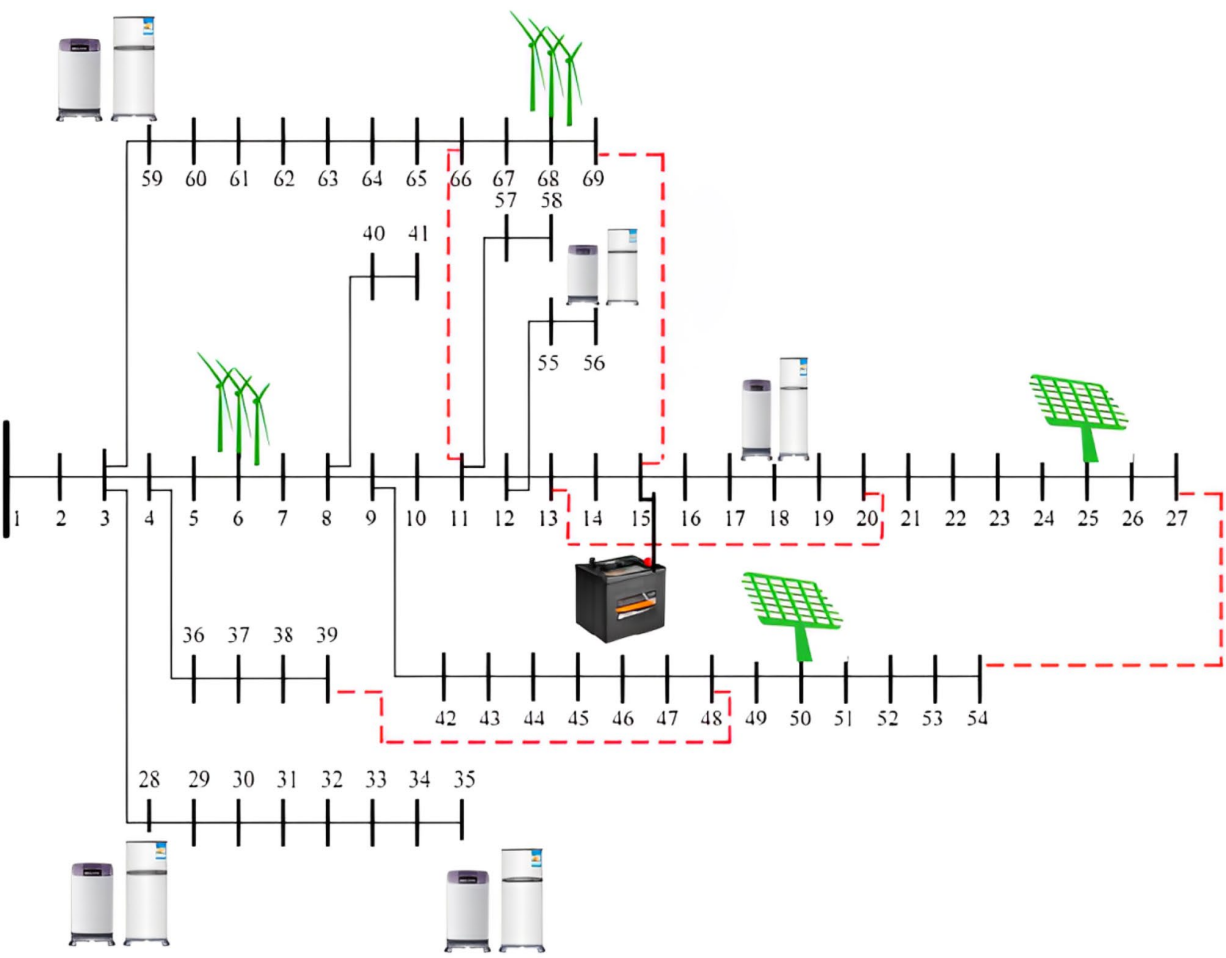
IEEE grid system^41^.

**Figure 4:**
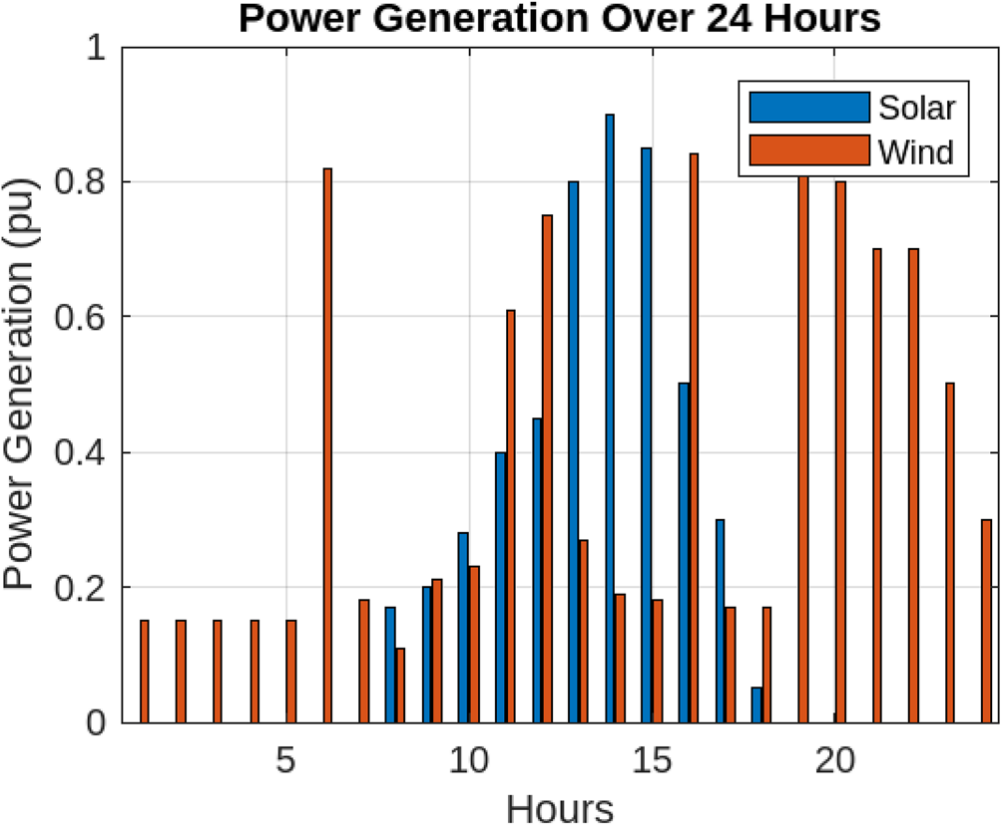
Renewable energy sources generation.

To evaluate the performance of the proposed machine learning model, the IEEE Microgrid test system is used, which is shown in Fig. 3. A Microgrid combines two wind turbines, two solar panels and a separate switch. Hybrid electric vehicles (HEVs) are placed evenly throughout the network, enabling charging based on their probability distribution, but equally across all buses. Connections connect the vehicle and provide flexibility for reconfiguration through 73 of the built-in components, 68 of which are separable. The Microgrid derives a large part of its electricity load from renewable sources, namely wind and solar power. The wind turbines of 142 and 250 kW follow the comparative efficiency model, which is demonstrated by the wind turbines of 200 and 180 kW to simplify the presentation. The raw data in Table 3 provides an overview of the total HEV charging needs and shows the unpredictability of HEV charging needs. The purpose of the proposed model, as described in Section 3, is to intelligently or collectively predict and distribute the total charge on the target day of operation. Figure 3 shows the battery storage units in the Microgrid, defined in Table 4. Four adjustable loads are taken into account within the Microgrid, with certain loads exhibiting shiftable characteristics defined in Table 5. Over a six-month period, historical data on the total energy charging demand of HEVs in the test system is utilized to train the proposed models .Parameters undergo adjustment and fixation through the Modified Differential Evolution Algorithm (MDA). Consequently, the proposed models provide predictions for the total charging demand of HEVs over a 24-hour cycle. Figure 4 depicts the Renewable Energy Sources Generation profile over a 24-hour period, emphasizing the dynamic output of solar and wind energy, showcasing their fluctuations and contributions to the overall energy generation throughout the day.To ensure fairness in comparison, the prediction period extends to 20 days, encompassing established methods such as ARMA, ANN, SVR, along with the proposed technique Krill defined in Table 6. The accuracy of predictions is then assessed, with an evaluation based on three key criteria: Mean Absolute Percentage Error (MAPE), Mean Absolute Relative Percentage Error (MARPE), and Root Mean Square Error (RMSE). (ARMA) exhibits moderate performance with a MAPE of 2.09 %, indicating reasonably accurate predictions, but it falls short in comparison to the precision achieved by Krill algorithm. Artificial Neural Network (ANN) and Support Vector Regression (SVR) showcase respectable performance, yet their MAPE values of 2.26% and 1.37% respectively suggest a marginally higher level of prediction error compared to the superior accuracy achieved by the krill Algorithm in this context.

The introduction of the Krill Herd Algorithm (KHA) to the prediction landscape reveals promising outcomes, with a MAPE of 1.02%. This underscores the algorithm’s effectiveness in predicting HEV charging demand.

**Figure 5:**
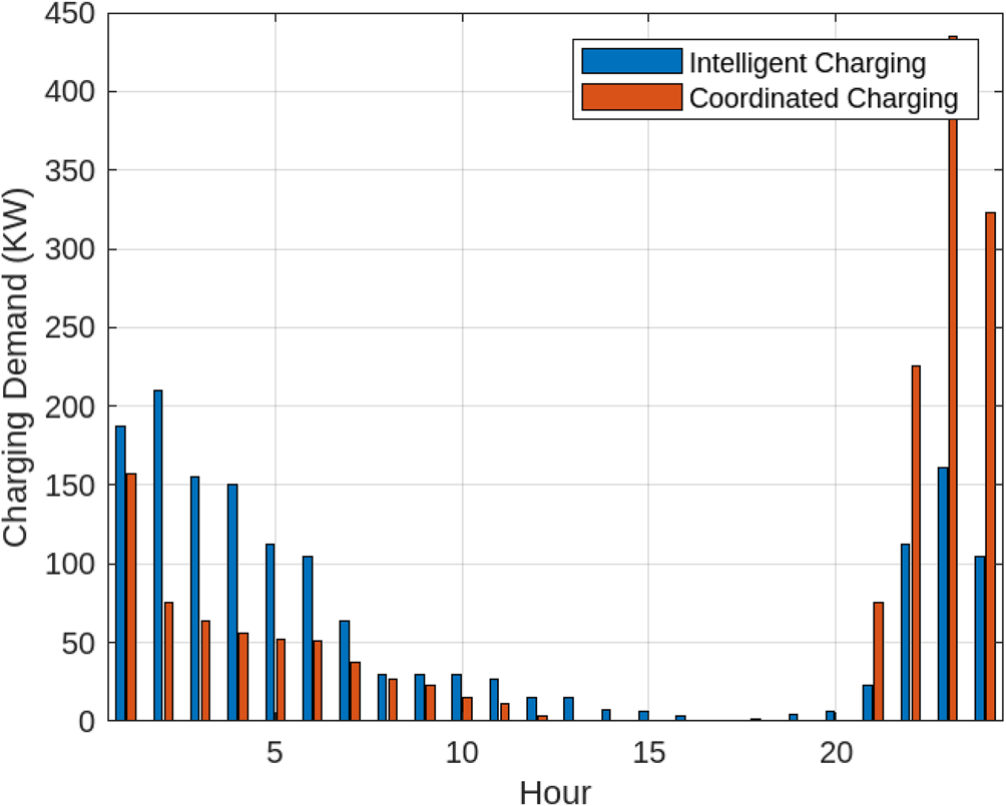
Comparative analysis of microgrid operation: coordinated vs. intelligent charging strategies with Krill optimization.

**Figure 6:**
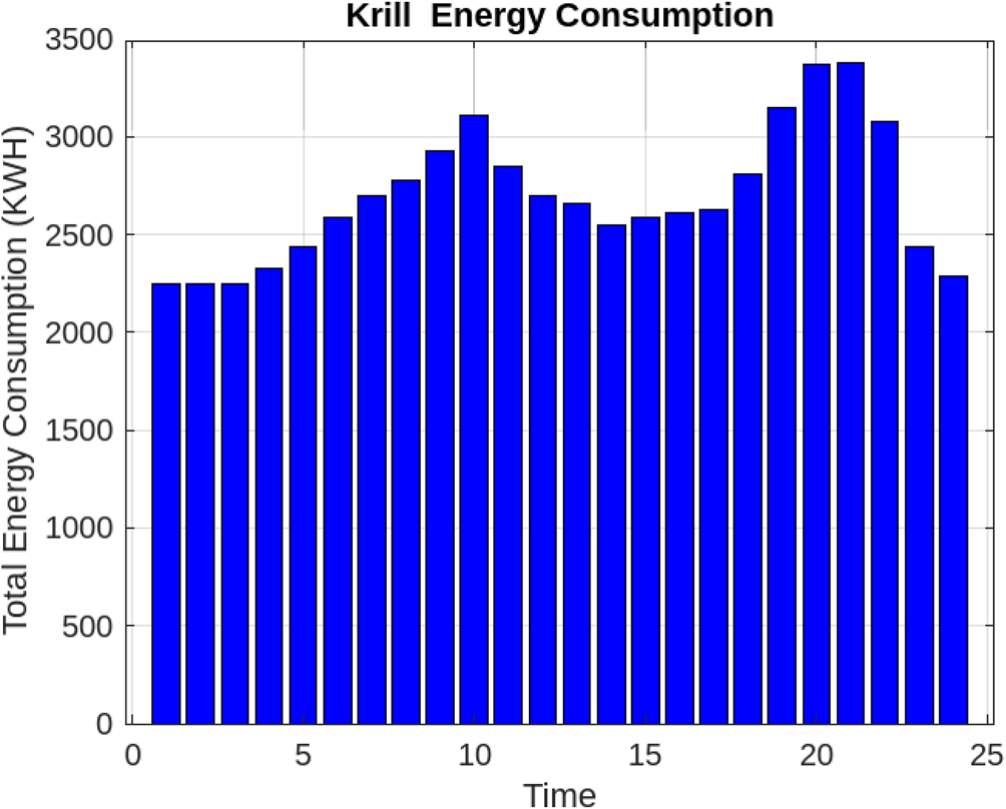
Energy consumption in microgrid operations resulted from through krill.

**Figure 7:**
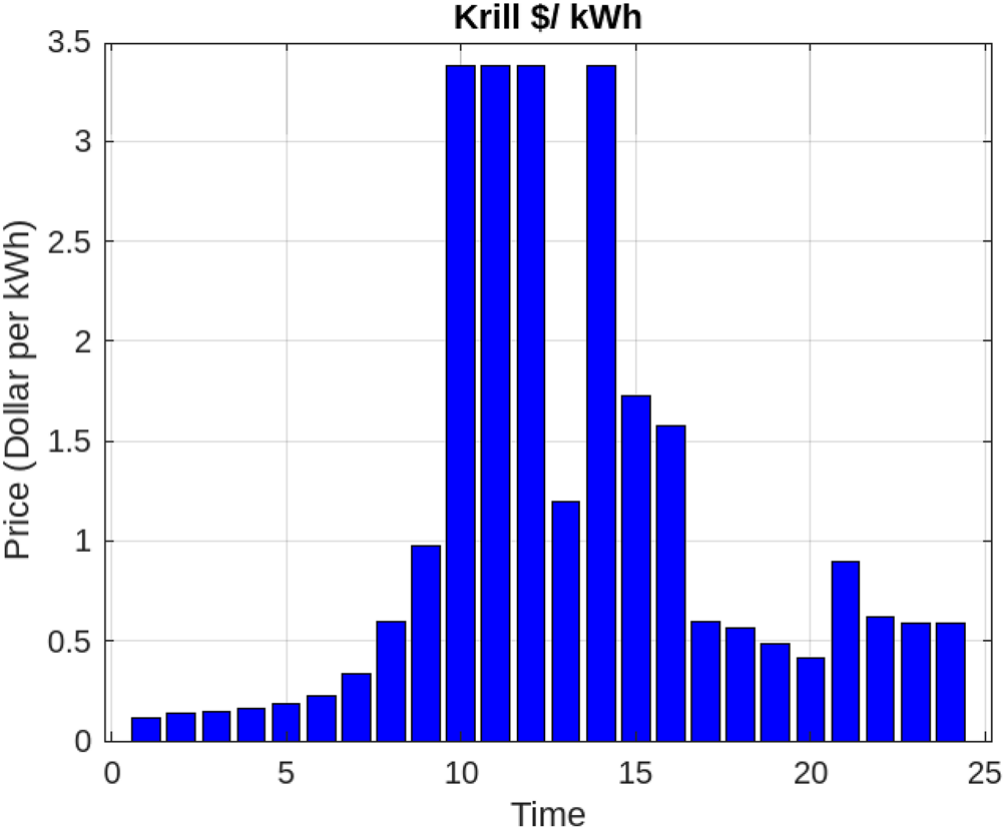
Krill market dynamics: hourly price variations in microgrid operations.

**Figure 8:**
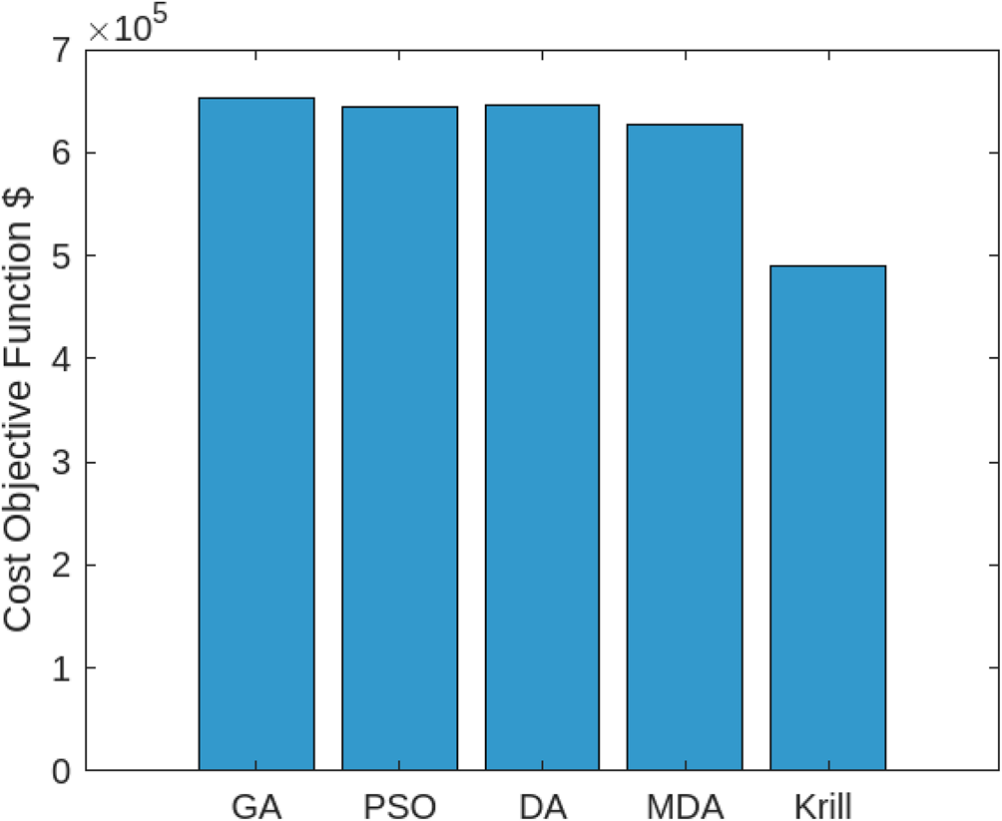
Performance analysis: convergence patterns of diverse optimization strategies for total cost function optimization.

**Figure 9:**
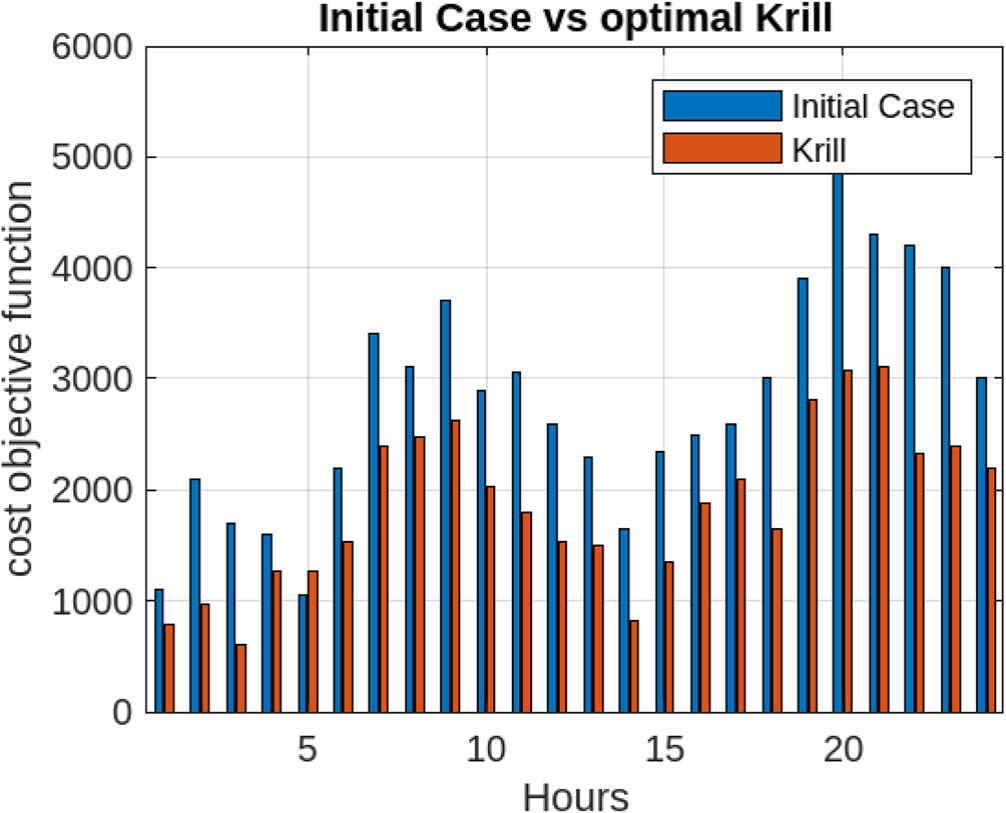
Hourly expenditure of the microgrid pre and post optimal switching operations through krill.

**Figure 10:**
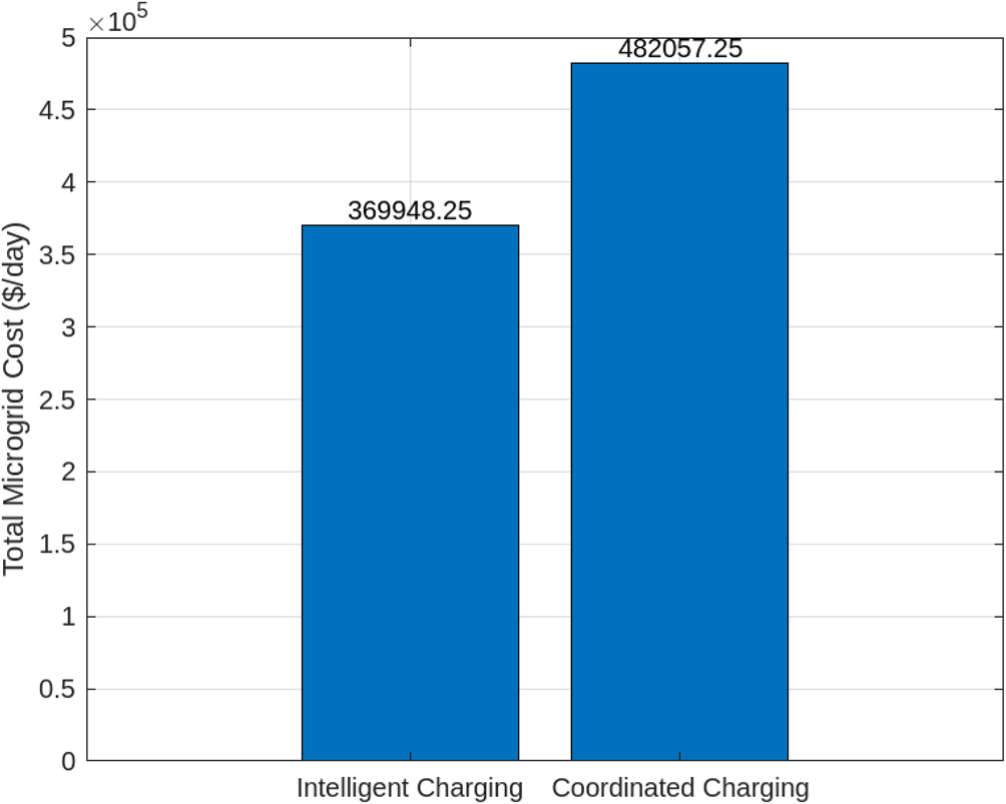
Relative operational expenses in coordinated and intelligent charging scenarios for microgrid.

**Figure 11:**
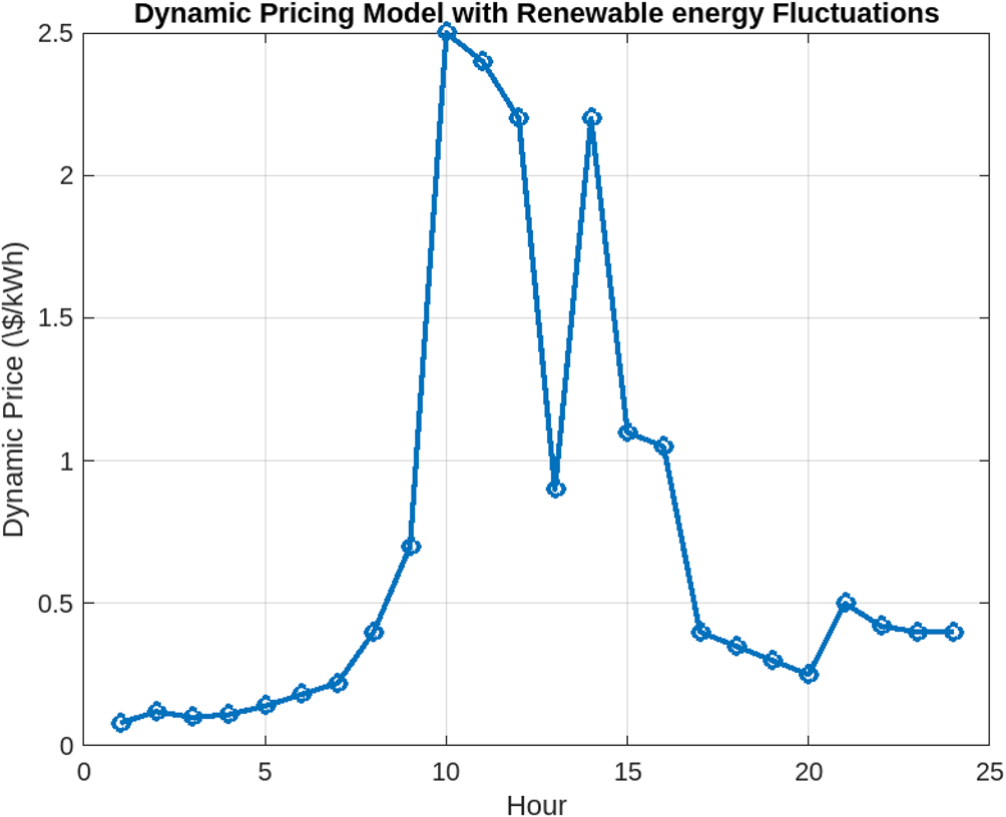
Dynamic fluctuation model.

**Figure 12:**
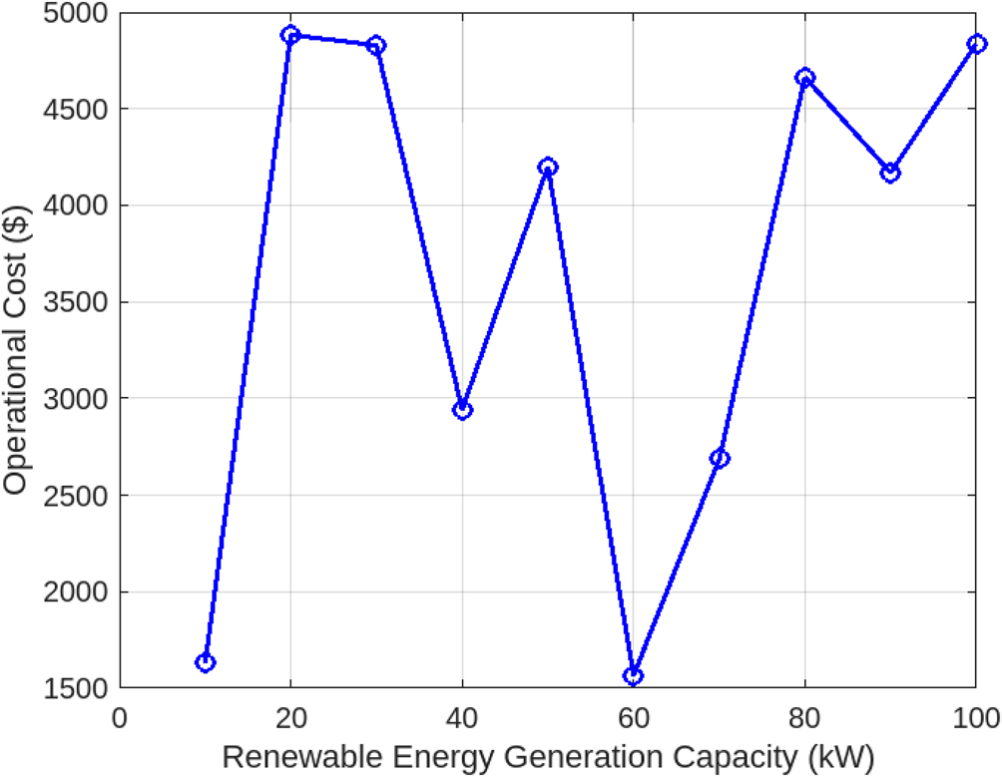
Sensitivity analysis: impact of renewable energy generation capacity.

Assessing the system’s performance at peak load hours involves a comparison of different charging strategies, distributing total load demand through coordinated and intelligent approaches. Figure 5 visually presents the HEV charging demand curves for the Krill algorithm.It illustrate energy consumption patterns for both Intelligent Charging (IC) and Coordinated Charging (CC). It is noteworthy that coordinated charging consistently demonstrates lower power consumption than intelligent charging throughout the entire 24-hour period, indicating its effectiveness in promoting a balanced and sustainable energy utilization strategy. The krill technique exhibits remarkable efficiency, illustrated by coordinated charging consuming 430 KW compared to 160 KW for intelligent charging during the peak consumption hour. The depicted optimization, as illustrated in Fig. 6, underscores the effectiveness of Krill in managing energy output, showcasing its capability to efficiently adapt and respond to diverse demands within the specified range from 2100 KWH to a maximum capacity.The proposed strategy keep consistently demonstrates lower power consumption, ranging. This consistent superiority in minimizing power usage suggests that krill Reduction is more effective in optimizing Microgrid cost compared to classical techniques. Figure 7 illustrate the hourly market prices for the Microgrid under the proposed technique.The time-dependent pricing unveils varying cost structures over the 24-hour period. Krill exhibits fluctuating prices, reaching a peak at 3.375/kWh during hours 10, 11, and 12, indicating a potential high-cost period. To assess the efficiency of the proposed algorithms in optimizing energy management, we conduct a comparative analysis with Particle Swarm Optimization (PSO), Genetic Algorithm (GA), and other proposed techniques. Table 7 presents cost and CPU time results for 24 hours over 40 trials, showcasing krill’s superior performance. Notably, even krill’s worst solution outperforms the best solutions from other systems, demonstrating robust optimization capabilities. The close alignment of the average index with the best solution underscores the reliability of the optimization process. Additionally, krill exhibits efficient computational performance, achieving optimal power sharing and unit scheduling in less time with minimal efforts. The lower standard deviation further emphasizes the robustness of the proposed algorithm. Remarkably, krill excels in CPU time efficiency, reducing it from 17.269 seconds to 14.248 seconds. This significant improvement highlights the computational advantages of the krill algorithm, ensuring faster convergence for optimal microgrid management. Moreover, its consistent cost-effectiveness, with a competitive cost profile compared to alternative algorithms, reinforces its reliability and efficiency in real-world applications. Figure 8 illustrate the total cost of the different optimization techniques.It can be noted from the figure and Table 7 that the krill outperforming other algorithms, which appear to become ensnared in local optima. Table 8 illustrates the optimized output power for the solar panel, wind turbine, and charging demand of the HEVs over a 24-hour period, as determined by the proposed method. The outcomes indicate that the renewable units are consistently generating power in accordance with the anticipated results.To enrich the comparative analysis, Fig. 9 vividly portrays the microgrid’s hourly cost over a 24-hour period, both before and after the implementation of network reconfiguration. The visual representation underscores the substantial benefits of network reconfiguration, showcasing remarkable reductions in losses and improved unit dispatch efficiency for the krill optimization technique. Moreover, the system consistently registers lower costs across each hour, underscoring the pivotal role of the switching process. These findings solidify the efficacy of the proposed model in optimizing renewable microgrids. Tables 9 unveil the optimal scheduling of the storage unit and adjustable loads in the microgrid for the three proposed techniques. In Table 9, the battery showcases a charging pattern in the early hours, followed by discharge in later hours, leading to a reduction in overall microgrid operation costs. This cyclic charging and discharging in the afternoon underscore the effectiveness of this strategy, with adjustable loads aptly scheduled within available time slots. Figure 10 compares the microgrid operation costs for intelligent charging and coordinated charging of HEV power demand. Intelligent charging, shifting demand to off-peak hours, consistently outperforms coordinated charging, resulting in lower costs 369,948.25 vs. 482,057.25. This strategy not only reduces total microgrid costs but also improves technical aspects, such as voltage profiles and alleviation of potential feeder congestion. The numbers highlight the economic advantage of intelligent charging, emphasizing its role in enhancing overall microgrid efficiency and financial viability. After determining the superior performance of the krill algorithm, a dynamic pricing model was developed to evaluate the system’s efficacy under renewable energy fluctuations. Applying the krill algorithm to this model yielded a responsive pricing structure, fluctuating between 0.08/kWh and 2.5/kWh throughout the day (shown in Fig. 11). This numerical illustration highlights the algorithm’s ability to adapt pricing dynamically to hourly variations, confirming its effectiveness in optimizing cost considerations. Furthermore, to comprehensively analyze the microgrid system’s performance, a sensitivity study assessed how variations in renewable energy generation capacity impact operational costs. This systematic approach explored different scenarios, revealing insights to optimize economic efficiency in real-world implementations. The analysis unveiled a non-linear relationship between renewable energy capacity and operational costs: initially, costs decreased gradually with greater reliance on renewables, but beyond a threshold, diminishing returns were observed (see Fig. 12). The insights gained from this sensitivity test lay the groundwork for future implementation and testing of our sustainable approach and represent a crucial step towards empirical validation in real-world environments. While the simulation environment adopted in this study is comprehensive, it may not fully encompass the intricacies of real-world complexities. The next critical step in our research agenda involves an in-depth investigation and empirical testing of this optimization approach in practical applications. This transition from simulation to real-world experimentation is imperative to validate and refine the proposed methodologies, ensuring their robustness and applicability in diverse operational settings. By conducting empirical testing, we aim to bridge the gap between theoretical concepts and practical implementation, ultimately advancing the field of renewable microgrid management.

**Table 3:** Historical charging demand data for HEVs (MWh).

Time (Day)	Charging demand (MWh)
1-8	2.3928, 2.1667, 2.2810, 2.3690, 2.4564, 2.4876, 2.2998, 2.1136
9-16	2.1185, 2.1678, 2.4336, 2.1663, 2.4215, 2.1614, 2.4739, 2.2099
17-24	2.1401, 2.1649, 2.3312, 2.2661, 2.2107, 2.4290, 2.3172, 2.3010
25-30	2.4684, 2.1807, 2.3955, 2.3939, 2.2238, 2.2783

**Table 4:** Characteristics of energy storage.

Storage	Bus	Capacity (kWh)	Min-Max charging/Discharging power (kW)
Name			Min charging/Discharging time (h)
DES	15	1500	50-250

**Table 5:** Load characteristics (S: Shiftable, C: Curtailable).

Load type	Bus	Capacity (kW)	Energy (kWh)	Time (h)	Min up time (h)
L1 S	28	0-60	240	11-14	1
L2 S	56	0-60	240	15-19	1
L3 S	18	20-60	240	16-19	1
L4 C	35	10-40	200	1-24	24
L5 C	59	20-60	300	13-24	12

**Table 6:** Performance metrics for different methods.

Method	MAPE (%)	MARPE	RMSE
ARMA	2.08753	6.25845	2.63238
ANN	2.26421	6.56019	2.86453
SVR	1.36508	3.40989	1.45778
GP_KHA	1.02381	104.64	0.78361

**Table 7:** Comparative analysis of optimization algorithms.

Method	Average	Worst	Best	S.D.	CPU time (s)
GA	6.6927	7.8865	6.5364	0.1524	17.269
PSO	6.5378	7.9124	6.4473	0.1325	15.703
DA	6.5085	7.5295	6.4527	0.1358	14.275
MDA	6.4163	6.5946	6.2653	0.1063	13.276
Krill	5.0195	5.8650	4.9023	0.0998	14.248

**Table 8:** Output power of wind units and solar panels along with the charging demands of HEVs.

Wind turbine 1 (kW)	Wind turbine 2 (kW)	Solar panel 1 (kW)	Solar panel 2 (kW)	HEV charging demand (Coordinate)	HEV charging demand (Intelligent)
16.898	29.7500	0	0	233.02	275.78
16.898	29.7500	0	0	106.52	289.04
16.898	29.7500	0	0	77.76	227.24
16.898	29.7500	0	0	72.00	210.00
16.898	29.7500	0	0	60.48	159.68
8.662	15.2500	0	0	50.40	136.68
16.898	29.7500	0	0	40.32	84.92
12.354	21.7500	1.6000	1.6000	28.80	53.28
16.898	29.7500	30.000	30.000	20.16	43.20
29.252	51.5000	60.200	60.200	11.52	41.74
83.070	146.250	83.600	83.600	8.64	38.86
98.548	173.5000	95.600	95.600	2.88	23.04
37.062	65.2500	191.20	191.20	1.44	20.16
22.436	39.5000	168.40	168.40	0	12.96
16.898	29.7500	63.000	63.000	0	7.20
12.354	21.7500	33.8000	33.8000	0	2.88
16.898	29.7500	4.4000	4.4000	0	0
16.898	29.7500	0	0	0	4.32
12.325	21.7000	0	0	0	17.26
16.898	29.7500	0	0	0	20.14
12.311	21.6750	0	0	113.6	50.34
12.311	21.6750	0	0	432.78	152.42
8.6620	15.2500	0	0	578.00	240.10
5.8220	10.2500	0	0	440.02	167.10

**Table 9:** Optimal switching pattern in the microgrid with a 25% improvement.

Storage	-1	-1	-1	-1	-1	1	1	1	1	1	-1	-1	-1	-1	-1	-1	1	1	1	1	1			
L1	0	0	0	0	0	0	0	0	0	0	1	1	1	1	0	0	0	0	0	0				
L2	0	0	0	0	0	0	0	0	0	0	0	0	0	1	1	1	1	1	0	0				
L3	0	0	0	0	0	0	0	0	0	0	0	0	0	0	1	1	1	1	0	0				
L4	1	1	1	1	1	1	1	1	1	1	1	1	1	1	1	1	1	1	1	1				
L5	0	0	0	0	0	0	0	0	0	0	0	1	1	1	1	1	1	1	1	1				
Hours	1	2	3	4	5	6	7	8	9	10	11	12	13	14	15	16	17	18	19	20	21	22	23	24
Sectionalizing switches	4	0	0	0	0	1	0	0	0	0	0	0	0	0	0	0	0	0	1	0	0	0		
5	0	0	0	1	0	0	0	0	0	0	0	0	0	0	0	0	0	0	0	1	0			
6	0	1	0	0	0	0	0	0	0	0	0	0	0	0	0	0	0	0	0	0	0			
7	0	0	0	0	0	0	0	0	0	1	0	0	0	1	0	0	0	0	0	0	0			
10	0	0	0	0	0	0	0	0	1	0	0	0	0	0	0	0	0	0	0	1	0			
11	0	0	0	0	0	0	1	0	0	0	0	0	0	0	0	0	0	1	0	0	0			
13	0	0	0	0	0	0	0	1	0	0	0	0	0	0	1	0	0	0	0	0	0			
18	0	0	1	0	0	0	0	0	1	0	0	0	0	0	0	0	0	1	0	1	0			
20	0	1	0	0	0	0	0	0	0	0	0	0	0	0	0	1	0	0	0	0	0			
23	0	0	0	0	0	0	0	0	0	0	0	0	1	0	0	0	0	0	0	0	0			
26	0	0	0	0	1	0	0	0	0	0	0	0	0	0	0	0	0	0	0	0	0			
29	0	0	0	0	0	0	1	0	0	0	0	0	0	0	1	0	1	0	0	0	0			
30	0	0	0	0	0	0	0	0	0	0	0	1	0	0	0	0	0	0	0	0	0			
33	1	0	0	0	0	0	0	0	0	0	0	0	1	0	0	1	0	0	0	0	0			
36	0	0	0	0	0	1	0	0	0	0	1	0	0	0	0	0	0	0	0	0	0			
38	0	0	1	0	0	0	0	0	0	0	0	0	0	0	0	0	0	0	0	0	0			
47	0	0	0	0	1	0	0	0	0	0	0	0	0	0	0	0	0	0	0	0	0			
49	0	0	0	0	0	0	1	0	0	0	1	0	0	0	1	0	0	0	0	0	0			
52	0	0	0	0	0	0	0	0	1	0	0	0	0	0	0	0	0	0	0	0	0			
57	0	0	0	0	0	0	0	0	0	0	1	0	0	0	0	0	0	0	0	0	0			
Tie switches	69	1	0	1	0	1	0	1	1	1	0	0	1	1	1	0	1	1	1	1	0	1		
70	0	1	0	1	1	0	0	1	1	1	1	1	0	0	1	0	0	0	1	1	1	0		
71	1	1	1	1	1	1	1	0	0	1	1	0	1	1	1	0	1	1	1	1	1	1		
72	1	0	0	1	0	1	1	1	1	0	1	0	1	0	1	0	0	1	0	1	0	1		
73	1	1	1	1	0	1	0	1	0	1	1	0	1	1	1	1	1	1	1	1	1	0		

The provided tables illustrate the optimal switching patterns in a microgrid, detailing the activation states of Sectionalizing and Tie Switches across 24 hours. These patterns are integral for managing the microgrid’s power distribution efficiently.In comparing Krill Harmony and Firefly optimization methods, it’s important to consider the trade-offs. Firefly optimization demonstrates superior performance in terms of minimizing Root Mean Square Error (RMSE) and Mean Absolute Deviation (MAD), indicating higher precision and accuracy in optimization results. However, Krill Harmony may offer cost advantages in terms of the objective function.

## Conclusion

In summary, this study introduces an innovative machine learning approach to address challenges in managing hybrid electric vehicle (HEV) charging demand within renewable microgrids. Utilizing Advanced Random Forest Regression models, the research shows significant improvements in Mean Absolute Percentage Error (MAPE), highlighting the precision of predictive models. The exploration of coordinated and intelligent charging scenarios, supported by nature-inspired optimization methods like the Gaussian Process Model and Krill Herd Algorithm, offers valuable insights for optimizing system efficiency. The self-adaptive modification mechanism enhances the optimization approach’s adaptability to diverse system characteristics, demonstrating its efficacy. Particularly noteworthy is the proposed modification for improved cost-effectiveness, as indicated by the detailed cost performance table. These findings contribute significantly to the field of renewable microgrid management, providing a practical and efficient framework for real-world applications. Moreover, the research not only addresses identified gaps in existing literature but also paves the way for further advancements in sustainable energy management. The seamless integration of machine learning, optimization algorithms, and adaptive strategies positions this study as a valuable resource for researchers, practitioners, and policymakers aiming for resilient and efficient renewable microgrid systems. The next step involves real-world implementation to validate the proposed methodologies, bridging the gap between simulation and reality for successful integration into the dynamic landscape of sustainable energy systems.To advance future research in sustainable energy management, a comprehensive plan is outlined. Firstly, exploration of advanced energy storage technologies will fortify microgrid resilience and efficiency. Secondly, investigation into the impact of regulatory and policy changes on microgrid operations will be conducted, acknowledging the evolving energy governance landscape. Additionally, integration of artificial intelligence into dynamic pricing models is proposed to optimize consumer behavior and overall microgrid performance. Prioritizing these areas will guide future research efforts and foster collaboration within the research community, thus advancing sustainable energy management practices. Following the verification of the proposed technique’s effectiveness through simulation results, a strategic plan will be executed to establish partnerships with microgrid operators and research institutions. Collaborative pilot projects, tailored with clear objectives and timelines, will integrate the model into selected microgrid sites representing diverse operational environments. Rigorous data collection and stakeholder feedback will drive an iterative refinement process, validating the model against historical data. This meticulous approach aims to bridge the gap between theoretical concepts and practical implementation in microgrid management.

## Data Availability

Te datasets used in/or analyzed during the current study are available from the corresponding author upon reasonable request.
